# Disparities in Advance Care Planning Across Rurality, Sociodemographic Characteristics, and Cognition Levels: Evidence from the Health and Retirement Study

**DOI:** 10.3390/jal4040028

**Published:** 2024-11-26

**Authors:** Zahra Rahemi, Juanita-Dawne R. Bacsu, Sophia Z. Shalhout, Morteza Sabet, Delaram Sirizi, Matthew Lee Smith, Swann Arp Adams

**Affiliations:** 1 School of Nursing, College of Behavioral, Social and Health Sciences, Clemson University, Clemson, SC 29634-0743, USA; 2 School of Nursing, Thompson Rivers University, Kamloops, BC V2C 0C8, Canada; 3 Department of Otolaryngology-Head and Neck Surgery, Harvard Medical School, Boston, MA 02115, USA; 4 Mike Toth Cancer Research Center, Boston, MA 02114, USA; 5 Computing and Applied Sciences, School of Mechanical and Automotive Engineering, College of Engineering, Clemson University, Clemson, SC 29634-0743, USA; 6 Department of Health Sciences, College of Behavioral, Social, and Health Sciences, Clemson University, Clemson, SC 29634, USA; 7 Department of Health Behavior, School of Public Health, Center for Community Health and Aging, Texas A&M University, College Station, TX 77843, USA; 8 Department of Epidemiology & Biostatistics, College of Nursing, University of South Carolina, Columbia, SC 29208, USA

**Keywords:** advance care planning, aging, cognition, disparities, ethnicity, race, social determinants of health

## Abstract

**Background:**

We aimed to examine ACP in older adults in the U.S. across different sociodemographic characteristics and cognition levels (N = 17,698).

**Methods:**

We utilized two legal documents from the Health and Retirement Study survey: a living will and durable power of attorney for healthcare (DPOAH). We established the baseline trends from 2014 to assess if trends in 2024 have improved upon future data availability. Logistic regression models were fitted with outcome variables (living will, DPOAH, and both) stratified by cognition levels (dementia/impaired cognition versus normal cognition).

**Results:**

Age, ethnicity, race, education, and rurality were significant predictors of ACP (having a living will, DPOAH, and both the living will and DPOAH) across cognition levels. Participants who were younger, Hispanic, black, less educated, or resided in rural areas were less likely to complete ACP.

**Conclusion:**

Examining ACP and its linkages to specific social determinants is crucial for understanding disparities and developing effective educational and interventional strategies to enhance ACP uptake among diverse population groups. Future studies are needed to assess whether disparities have improved over the last decade, particularly as 2024 data become available. Addressing ACP disparities is essential for healthcare professionals to advance research and promote effective practices in geriatric care and aging services.

## 1. Introduction

Among adults aged 65 and older in the United States (U.S.), Alzheimer’s disease and related dementias (ADRD) are the fifth leading cause of death. The prevalence of ADRD in the U.S. is projected to reach approximately 14 million by the year 2050 [1]. The prevalence and challenges of ADRD differ by ethnicity [2], rurality [3,4], gender, education, age, and co-morbidities [5]. For instance, this prevalence is elevated among individuals of Hispanic ethnicity, residing in rural areas, identifying as female, having lower levels of education, being of older age, and having co-morbidities. Overall, older adults from underrepresented and racial/ethnic minority groups have higher rates of chronic disease and cognitive impairment and lower rates of advance care planning (ACP) and comfort care at the end of life (EOL) [3,6]. It is imperative to explore the underlying causes of disparities in ACP and EOL care, aiming to eradicate inequities in healthcare based on factors such as race, ethnicity, and other personal identities.

ACP is a process across different levels of health and encompasses both formal mechanisms or written documents—such as advance directives, living will, and durable power of attorney for healthcare (DPOAH)—and informal mechanisms—including verbal discussions of healthcare preferences with family and providers [7–9]. ACP facilitates proactive communication of healthcare decisions before impairment of decision-making capacity. The goal of ACP is to ensure that care aligns with the individual’s preferences and goals, regardless of what those preferences may be, and to promote shared decision-making. The evidence indicates that ACP improves the quality of care, communication satisfaction, and patient care satisfaction among patients, surrogates, and healthcare providers. ACP also reduces distress for surrogates and healthcare providers while promoting decision-making aligned with patients’ values and goals [8,9]. ACP holds particular significance for individuals experiencing cognitive impairment, as their gradual loss of decision-making capacity, along with issues related to EOL, may present challenges for both their family members and proxy decision-makers.

In the context of ADRD patients, ACP and EOL care present distinct challenges [6,10]. As dementia progresses and decisional capacity diminishes, healthcare decisions often fall to a family member or proxy. This can be particularly challenging when life expectancy remains uncertain and ACP discussions have not taken place [11]. Challenges also arise within the context of diverse sociodemographic characteristics, particularly among racial and ethnic minority groups. ACP is influenced by several key sociodemographic factors, including race, ethnicity, and rurality, which affect access to and engagement with ACP resources and services. Studies show that disparities in ACP uptake and outcomes exist for minority groups and rural populations, which underscores the need for tailored approaches in these communities [4,12,13]. For instance, individuals from racially/ethnically diverse backgrounds who have not completed ACP often report lacking a family member to designate as their proxy [12]. Incorporating such contextual factors in research studies can enhance understanding about ACP disparities and improve the alignment of ACP practices with diverse patient needs.

This study used the 2014 Health and Retirement Study (HRS) data to examine past ACP in U.S. older adults across different sociodemographic characteristics and cognition levels. Compared to its preceding waves, we chose the 2014 wave because it offered the most comprehensive data among the selected harmonized datasets, particularly in End-of-Life (EOL) sections. Our overarching objective is to replicate the study using the HRS 2024 data upon its release, enabling us to assess ACP disparities over a span of 10 years. We are particularly interested in understanding how ACP disparities may change over time. Utilizing HRS waves aids in the completion of promising ongoing studies and leverages long-term data to provide valuable insights for establishing the best practices in addressing health disparities, including topics related to EOL disparities. Additionally, there is limited research exploring ACP disparities in rural populations, particularly regarding cognitive levels within marginalized groups, which highlights the novelty of this study. In this study, using national HRS datasets, we aim to reveal disparities in ACP engagement among these populations. Understanding ACP disparities based on rurality and sociodemographic factors can help clarify access and decision-making challenges in diverse communities.

Existing studies document trends in disparities over time among marginalized groups in relation to ACP. More specifically, structural disparities in accessing ACP have included issues of institutional racism, implicit bias, practitioner discomfort in discussing EOL planning, language differences, lack of cultural awareness (different beliefs and values) [11], exacerbation of institutional barriers/constraints, and pre-conceived notions of patients’EOL wishes [14]. Moreover, healthcare providers are documented as avoiding ACP conversations with patients from racial and/or ethnic minority groups (such as black, Asian, Hispanic, and Native American), non-English speaking patients with low incomes, and individuals with low health literacy [11]. Individual-level issues related to disparities included lack of ACP awareness and knowledge, inability to make decisions; no surrogates available, patient discomfort in discussing ACP (religious, cultural, or individual factors), lack of trust in healthcare providers and the healthcare system [15], family involvement, financial challenges, and faith and religious beliefs [14]. Despite this knowledge, existing studies do not examine past ACP disparities in relation to different cognition levels. However, evaluating changes in ACP disparities over time among marginalized population groups, including different cognition levels is crucial for improving health equity and access to care, especially for people living with dementia.

We defined ACP as encompassing a living will and DPOAH. A living will is a document that articulates a person’s preferences for medical treatments, offering insight into the healthcare they wish to receive (or decline) during severe health conditions or when their medical decision-making capacity is impaired. A DPOAH designates a person to make healthcare decisions on behalf of another individual when they are incapable of making decisions themselves [9]. The theoretical framework underpinning this research is the end-of-life-care-planning model, a conceptual nursing model that provides a framework for understanding factors associated with ACP disparities in diverse individuals [16]. The end-of-life-care-planning model suggests that such planning is influenced by diverse factors across personal, stakeholder, and environmental domains [16]. Personal factors include sociodemographic characteristics and cultural context, while stakeholder influences span individuals, families, clinicians, and policymakers. Environmental factors encompass social networks, caregiving dynamics, and healthcare settings. For this study, we concentrated on personal and environmental elements, specifically examining variables such as education, age, gender, race, ethnicity, marital status, rurality, everyday discrimination, spousal and family social support, and loneliness. Our findings hold significance, offering insights that can inform strategies for healthcare professionals and policymakers to reduce ACP disparities and improve EOL care practices across diverse healthcare systems, ultimately enhancing geriatric care and aging services.

## 2. Materials and Methods

### 2.1. Design and Setting

We conducted an observational, cross-sectional study utilizing the HRS, a comprehensive nationally representative dataset encompassing information from over 43,000 respondents. The HRS team employs a probability sample method, with oversampling of African American, Hispanic, and Floridian participants, to gather information from adults aged 51 years and older every two years. This data collection covers various aspects, including health, functional status, family structure, demographics, and lifestyle activities [17].

Our analyses used a sample of 17,698 participants from the HRS 2014 survey (Wave 12). To ensure adequate statistical power and examine relationships between study variables, we combined data from two different HRS datasets to create a pooled cross-sectional dataset: the harmonized HRS version B and the 1992–2016 Rand HRS Longitudinal version 2. Sub-data files were generated from the original files, and subsequent procedures were performed, such as data cleaning, computing, and appropriate variable recoding (e.g., setting values like ‘refused’ or ‘don’t know’ to missing). The final dataset was then created by merging all subfiles.

### 2.2. Variables

Race was captured as white, black, or other. However, the ‘other’ category of race was statistically underpowered for analysis with the study outcomes and was excluded from the analysis. Ethnicity was designated as Hispanic versus non-Hispanic. Education was categorized as less than or equal to a high school education and greater than a high school education. Rurality was grouped as an urban or rural residence. Marital status was designated as married/partnered or not married/divorced/widowed/single. Variance Inflation Factor (VIF) was used to identify multicollinearity and to quantify how much the variance of a regression coefficient is inflated due to the linear relationship between each predictor and the other predictors in the model. The results indicated no significant collinearity, as all VIF values were below the threshold for problematic multicollinearity.

### 2.3. Data Analysis

Data analyses were performed using SAS Version 9.4. Descriptive statistics were computed to explore the frequencies and distributions of key variables. Given the HRS’s complex sampling design, which includes oversampling of minorities and multiple respondents from a household, individual and household weights were utilized for some analyses [18]. Descriptive statistics were analyzed using weighted methods (Proc SurveyMeans, Proc SurveyFreq) to account for survey design complexities. This also allowed us to calculate frequencies (uni- and bi-variable analyses) that are representative of the U.S. population. A previous work indicates that these same weights introduce bias to most multivariable regression models [19]; therefore, we employed unweighted regression modeling procedures (Proc Logistic) for these analyses.

The primary outcomes modeled were whether the respondents had a living will (“whether respondent has a living will” [1 = yes, 0 = no]) and a DPOAH (“whether respondent has durable power of attorney” [1 = yes, 0 = no]). A summary joint outcome variable was created from these two variables (having both ACP measures, at least one, and none). Predictor variables included age at interview, gender, ethnicity, race, education, marital status, rurality, everyday discrimination score, social support score (both spousal and friends), and loneliness score.

We adopted the Langa–Weir approach, a composite measure that ranges from 0 to 27, to distinguish cognitive impairment from more advanced dementia [20]. The HRS employs a comprehensive approach to assessing dementia and cognitive function, utilizing objective cognitive tests (actual cognitive assessments) rather than subjective survey responses to ensure accurate and reliable data. In this study, the cohort was split into two groups: dementia/impaired cognition (Langa–Weir score ≤ 11) and normal cognition (Langa–Weir score > 11). Logistic regression models were run with each outcome variable (having living will, DPOAH, and both living will and DPOAH) stratified by cognition (dementia/impaired cognition versus normal cognition). Since the combination variable (both living will and DPOAH) had 3 levels, we utilized polytomous logistic regression models. In the full model for each outcome, we incorporated all predictor variables that were significant in the univariate models. For all variables (both independent and dependent), any value that was indicated as ‘refused’, ‘not answered’, or not recorded was set to missing for all analyses. Because we had a more-than-ample sample size, no imputations were performed to attempt to assign values for missing data. Moreover, imputation was not performed because the investigators were concerned that doing so would introduce unnecessary bias into the analyses. Consequently, all multivariable models only included those participants with no missing values.

## 3. Results

Of the 17,698 respondents, 77.8% had normal cognition, and 22.2% had a diagnosis of dementia/impaired cognition. Participants who were older, Hispanic, black, single/widowed (no partner), had lower levels of education, and had higher loneliness scores were more likely to have dementia/impaired cognition than their counterparts (see Table 1). Those with impaired cognition or dementia had significantly higher spousal support compared to those without dementia (*p* < 0.01) but significantly lower other family social support (*p* < 0.01). Additionally, among those with dementia or impaired cognition, only 46.2% had a living will, compared to 54.2% among normal cognition individuals (*p* < 0.01). Similarly, only 50.6% of those individuals with dementia or impaired cognition had a durable power of attorney versus 54.2% of those individuals with normal cognition (*p* < 0.03).

Among those with dementia or impaired cognition, 38.7% had both a DPOA and living will, compared to 47.5% among normal-cognition individuals (*p* < 0.01).

The significant predictors of having a living will, stratified by cognition status, are shown in Table 2. For those individuals with dementia or impaired cognition, race, ethnicity, education, and age and age were associated with having a living will such that black persons were 0.36 (95% CI: 0.21, 0.61) times less likely, Hispanic persons were 0.34 (95% CI: 0.19, 0.63) times less likely, and those with more than a high school education were 2.27 (95% CI: 1.43, 3.62) times more likely. The odds of having a living will increased by 7% for each 1 year increase in age among persons with dementia or impaired cognition. Among those with normal cognition, race (OR_black_ = 0.43, 95% CI: 0.30–0.63), ethnicity (OR_Hispanic_ = 0.30, 95% CI: 0.19–0.47), rurality (OR_rural_ = 0.81, 95% CI: 0.65, 0.99), education (OR_>HS_ = 1.85, 95% CI: 1.51–2.25), and age (OR = 1.07, 95% CI: 1.05–1.09) were significant predictors of having a living will.

Similar findings were demonstrated for DPOA (Table 2). Among individuals with dementia or impaired cognition, only education (OR_>HS_ = 1.96, 95% CI: 1.25–3.06) and age (OR = 1.05, 95% CI: 1.02–1.08) were significant predictors of DPOA. Among those with normal cognition, race (OR_black_ = 0.64, 95% CI: 0.44–0.92), ethnicity (OR_Hispanic_ = 0.43, 95% CI: 0.28–0.66), education (OR_>HS_ = 1.85, 95% CI: 1.52–2.26), and age (OR = 1.07, 95% CI: 1.05–1.08) were significant predictors.

In models examining the joint effects of having a living will or DPOA, higher education and age significantly predicted increased odds of having both or at least one ACP for both dementia/impaired-cognition individuals and normal-cognition individuals (Table 3). Black race and Hispanic ethnicity predicted a lower likelihood of having both ACP measures but not just one ACP for both cognition groups. Living in a rural area was only a significant predictor of the decreased likelihood of having both ACP measures among normal cognition individuals.

## 4. Discussion

We examined the involvement of older adults in ACP (having a living will and DPOAH) and identified the factors associated with ACP among participants with different cognitive statuses. Consistent with the end-of-life-care-planning model [16], our findings revealed ACP disparities based on sociodemographic factors and cognition levels. Individuals living with cognitive impairments and with specific identities, such as Hispanic and black participants, those with lower education levels, and residents of rural areas, had lower rates of ACP. Our results showed ACP disparities among diverse populations.

Cognitive impairments and ADRD are progressive conditions, and patients gradually lose their decision-making ability and cognitive function. Therefore, early healthcare decision-making and planning through ACP can significantly enhance the quality of care and reduce stress and anxiety for individuals with cognitive impairments and caregivers [8,21]. Moreover, ACP’s proactive approach reduces anxiety about last-minute decisions for patients and their caregivers [8,22]. There are multiple barriers to initiating ACP in individuals with ADRD, including topic avoidance, stigma/fear of diagnosis, denial and family disagreements, inadequate knowledge, ADRD behavioral changes, dementia trajectory, and nihilistic views that nothing can be done [6,11,23–25]. Additionally, research suggests that ACP barriers encompass various sociodemographic factors, living and residential conditions, acculturation, spirituality, religiosity, absence of a proxy for an appointment if required, and cultural beliefs [26–29]. Consistent with our results, racial/ethnic minority, culturally diverse, and rural populations experience disparities in ACP and EOL care [4,27,28]. Engagement in ACP is less common among minority populations, including African Americans, Hispanic Americans, and Asian Americans, and they are more inclined to prefer intensive and non-beneficial life-sustaining interventions over comfort care at EOL [28,29].

To improve ACP, it is essential to understand the diversity of planning needs and the sociodemographic and cultural backgrounds of individuals. Adoption of ACP has been limited, despite its well-established benefits for patients, families, and society [4]. Barriers to adoption include challenges in initiating conversations, documenting plans, and following up, with environmental context, professional role, and emotional factors playing significant roles in influencing ACP uptake, especially in rural healthcare settings [10]. Research has increasingly highlighted these disparities and used them to develop more person-centered approaches to ACP. For example, a family-centered ACP model adapted to Asian cultural values was found to improve dementia care understanding and reduce decisional conflict for both patients and caregivers, particularly with the use of visual aids [30]. This intervention underscores the importance of family involvement and structured facilitation in ACP, especially for those with dementia. Similarly, studies in rural areas have shown that smaller healthcare networks, trusted relationships, and the tight-knit nature of rural communities can serve as facilitators for ACP adoption in these settings, further emphasizing the need for culturally sensitive and context-driven approaches [4,30].

Research indicates that the lack of ACP can lead to reduced utilization of supportive care, higher rates of non-beneficial life-sustaining interventions at EOL, increased stress for caregivers, and diminished care satisfaction [8,11,22]. ACP is crucial to enhancing shared decision-making and facilitating discussions about EOL care options, including hospice services. It enables care management that aligns with the patient’s and their family’s preferences, contributing to improved patient dignity [4,31]. Improving ACP engagement is especially important for people with cognitive impairment before they lose functional, cognitive, and decisional capacity. Research and comprehensive communication about ACP and shared decision-making among people with cognitive impairment is crucial, especially considering recent debates within the palliative care community about the utility of ACP and its desired effects [32]

This study focused on understanding ACP disparities among diverse participants across different cognition levels and identities in the past to serve as a baseline for comparison today upon release of the 2024 data. This will allow us to determine improvements, if any, in trends. Our findings may further EOL-related research, practice, and policy by informing the development of dementia-specific and person-centered ACP methods. The results can incorporate the body of knowledge to be used for reducing racial and ethnic disparities related to ACP and EOL care.

This study contributes to the field by examining disparities in ACP engagement between different groups, especially in rural versus urban populations, an area often over-looked in previous research. Using an end-of-life care framework, this research uniquely addresses the intersection of sociodemographic diversity and cognitive impairments—a critical yet underexplored aspect of ACP. These findings underscore the importance of developing tailored approaches within diverse populations, which aligns with the framework’s emphasis on personalized, inclusive ACP practices [7].

This study’s findings provide crucial insights into the rurality and sociodemographic disparities in ACP engagement, including those with cognitive impairments. Given the scarcity of research in these areas, the results highlight the need for more culturally and cognitively appropriate ACP tools that address the unique needs of these groups.

For future research, it is critical to focus on developing tailored interventions that address ACP disparities, ensuring that diverse populations, including marginalized and minority groups, have the resources and support they need for end-of-life decision-making. In practice, healthcare providers should receive training in culturally competent and cognitively accessible ACP methods. Policymakers are urged to incorporate these findings into guidelines and interventions, aiming to reduce ACP disparities and improve healthcare access and quality for all populations.

### Limitations

Our study was limited to secondary data analyses, as it drew from existing data of U.S. adults and measurement tools. However, a strength of the study was its reliance on a nationally representative survey of adults, which minimized the potential for sampling bias and threats to external validity. The HRS, with an oversampling of black and Hispanic older adults, helps investigate and address racial and ethnic health disparities across the U.S. In addition, there are limitations that warrant discussion when using race as a variable in health disparity studies, including conceptual ambiguity around race, which is a socially constructed category rather than a biologically or genetically defined one. This may lead to inconsistencies in how race is defined, categorized, and interpreted in research. Race also may lead to internal validity concerns, obscuring the true relationship between race and the outcome of interest. Accurately measuring and categorizing race in a survey can be problematic, and the categories allowed may not capture the full complexity of individuals’ racial identities or experiences. Finally, it is important to note that race does not operate in isolation; it intersects with other social categories like gender, class, and ethnicity, and the interpretation of results should take the complexity of race under consideration.

## 5. Conclusions

Our study identified and quantified ACP disparities among older adults with cognitive impairment and diverse identities, particularly concerning ethnicity/race, rurality, and decreased educational attainment. These findings provide valuable information for shaping educational interventions that support EOL planning for diverse older adults. Understanding ACP barriers can aid healthcare providers in initiating proactive ACP discussions with patients living with ADRD and their caregivers, addressing disparities in ACP and EOL care based on race and ethnicity. Our findings can inform research, practice, and policy aimed at enhancing ACP for cognitively impaired populations. Future research should focus on developing and evaluating culturally and cognitively appropriate ACP interventions that address disparities across diverse populations, especially among rural, urban, and cognitively impaired groups. This study’s findings, framed by the end-of-life care model, underscore the importance of training healthcare providers in inclusive ACP practices and guiding policymakers in creating equitable ACP policies. The insights from this study can assist healthcare professionals in developing strategies to reduce disparities within marginalized and minority groups. As we await the release of 2024 data, it will be crucial to assess whether these disparities have improved over the last decade and deepen our understanding of the factors that perpetuate healthcare and EOL disparities.

## Figures and Tables

**Table 1.** Descriptive statistics for the 2014 Health and Retirement Survey cohort by cognition group (HRS 2014).

	Normal Cognition N = 13,774 Weighted % (n)	Dementia/Impaired Cognition N = 3924 Weighted % (n)	*p*-Value
Education			
≤ 12 years	38.8 (5572)	72.8 (2733)	<0.01
13+ years	61.2 (7062)	27.2 (890)	
Gender			
Male	45.1 (5304)	45.4 (1517)	0.05
Female	54.9 (7494)	54.6 (2124)	
Race			
White	86.3 (9666)	66.9 (2084)	<0.01
Black	13.7 (2999)	33.1 (1546)	
Ethnicity			
Hispanic	7.1 (1444)	17.0 (752)	<0.01
Non-Hispanic	92.9 (11237)	83.0 (2882)	
Marital Status			
Married/Living with a Partner	76.0 (9743)	70.4 (2708)	<0.01
Single/Widowed	24.0 (2952)	29.6 (931)	
Rurality			
Rural	26.4 (3163)	28.3 (937)	0.27
Urban	73.6 (9480)	71.7 (2678)	
Living Will			
Yes	54.2 (3743)	46.2 (1071)	<0.01
No	45.8 (3051)	53.8 (1396)	
Durable Power of Attorney			
Yes	54.2 (3787)	50.6 (1215)	<0.03
No	45.8 (3024)	49.4 (1252)	
Joint Effects			
Both DPOA and Living Will	47.5 (3259)	38.7 (892)	<0.01
Either DPOA or Living Will	13.3 (956)	19.1 (470)	
None	39.2 (2547)	42.2 (1078)	
	Mean (SE)	Mean (SE)	
Age (yrs)	65.7 (0.3)	72.3 (0.4)	<0.01
Everyday Discrimination Score	1.54 (0.01)	1.59 (0.02)	0.07
Spousal Social Support Score	1.72 (0.01)	1.86 (0.03)	<0.01
Other Family Social Support Score	1.82 (0.01)	1.76 (0.02)	<0.01
Loneliness Score	1.50 (0.01)	1.63 (0.02)	<0.01

Everyday Discrimination: Scores range from 1 to 6; a higher score indicates greater perceived discrimination. Spousal Social Support Score: Scores range from 1 to 4; a higher score indicates greater support. Other Family Social Support Score: Scores range from 1 to 4; a higher score indicates greater support. Loneliness: Scores range from 1 to 3; a higher score indicates greater perceived loneliness.

**Table 2.** Living will and durable power of attorney prediction models.

		Living Will				Durable Power of Attorney			
		Dementia/Impaired Cognition N = 517		Normal Cognition N = 1850		Dementia/Impaired Cognition N = 517		Normal Cognition N = 1844	
		Odds Ratio	95% CI	Odds Ratio	95% CI	Odds Ratio	95% CI	Odds Ratio	95% CI
Race	White	1.00		1.00		1.00		1.00	
	Black	0.36	**0.21, 0.61**	**0.43**	**0.30, 0.63**	0.69	0.43, 1.11	**0.64**	**0.44, 0.92**
Ethnicity	Not Hispanic	1.00		1.00		1.00		1.00	
	Hispanic	**0.34**	**0.19, 0.63**	**0.30**	**0.19, 0.47**	0.60	0.34, 1.04	**0.43**	**0.28, 0.66**
Rural	No	1.00		1.00		1.00		1.00	
	Yes	0.83	0.54, 1.27	**0.81**	**0.65, 0.99**	1.16	0.78, 1.73	0.82	0.66, 1.01
Marital Status	Single	1.00		1.00		1.00		1.00	
	Married	1.36	0.58, 3.21	0.88	0.55, 1.40	1.59	0.73, 3.46	0.95	0.60, 1.50
Gender	Male	1.00		1.00		1.00		1.00	
	Female	0.98	0.66, 1.44	1.13	0.93, 1.39	1.02	0.70, 1.48	1.17	0.96, 1.43
Education	≤HS	1.00		1.00		1.00		1.00	
	>HS	**2.27**	**1.43, 3.62**	**1.85**	**1.51, 2.25**	**1.96**	**1.25, 3.06**	**1.85**	**1.52, 2.26**
Age		**1.07**	**1.04, 1.11**	**1.07**	**1.05, 1.09**	**1.05**	**1.02, 1.08**	**1.07**	**1.05, 1.08**
Everyday Discrimination		1.14	0.86, 1.51	1.05	0.88, 1.26	0.93	0.72, 1.21	1.07	0.89, 1.27
Other Support		0.82	0.56, 1.20	1.02	0.84, 1.25	1.02	0.71, 1.45	1.05	0.86, 1.27
Spousal Support		1.01	0.68, 1.51	0.94	0.77, 1.15	0.98	0.67, 1.42	0.83	0.68, 1.01
Loneliness Score		1.16	0.69, 1.94	0.78	0.60, 1.02	1.30	0.80, 2.09	0.87	0.66, 1.13

Note. Results in bold indicate significance at a significance level of 0.05.

**Table 3.** Joint effects of living will and durable power of attorney prediction model.

		Dementia/Impaired Cognition N = 511		Normal Cognition N = 1844	
		OR (95% CI) Both LW/DPOA	OR (95% CI) Just One ACP *	Odds Ratio Both LW/DPOA	95% CI Just One ACP *
Race	White	1.00	1.00	1.00	1.00
	Black	**0.38 (0.21, 0.69)**	1.36 (0.74, 2.52)	**0.46 (0.30, 0.70)**	1.19 (0.73, 1.94)
Ethnicity	Not Hispanic	1.00	1.00	1.00	1.00
	Hispanic	**0.36 (0.18, 0.70)**	0.82 (0.39, 1.70)	**0.32 (0.20, 0.52)**	**0.40 (0.20, 0.82)**
Rural	No	1.00	1.00	1.00	1.00
	Yes	0.93 (0.58, 1.48)	1.36 (0.80, 2.32)	**0.79 (0.63, 0.99)**	1.12 (0.82, 1.53)
Marital Status	Single	1.00	1.00	1.00	1.00
	Married	1.73 (0.63, 4.75)	1.11 (0.44, 2.75)	0.90 (0.55, 1.48)	0.99 (0.49, 2.00)
Gender	Male	1.00	1.00	1.00	1.00
	Female	1.02 (0.66, 1.57)	0.79 (0.48, 1.30)	1.19 (0.96, 1.48)	1.18 (0.87, 1.60)
Education	≤HS	1.00	1.00	1.00	1.00
	>HS	**2.65 (1.56, 4.51)**	**2.61 (1.43, 4.79)**	**2.07 (1.67, 2.57)**	**1.37 (1.02, 1.85)**
Age		**1.08 (1.04, 1.12)**	**1.05 (1.01, 1.10)**	**1.08 (1.06, 1.10)**	**1.07 (1.04, 1.10)**
Everyday Discrimination Score		1.01 (0.73, 1.39)	1.29 (0.93, 1.78)	1.07 (0.88, 1.31)	1.17 (0.90, 1.51)
Other Support		0.87 (0.56, 1.33)	1.42 (0.90, 2.25)	1.04 (0.84, 1.30)	1.33 (0.99. 1.79)
Spousal Support		1.05 (0.68, 1.63)	0.60 (0.35, 1.03)	0.85 (0.68, 1.06)	0.89 (0.65, 1.21)
Loneliness Score		1.22 (0.69, 2.17)	1.43 (0.76, 2.68)	0.79 (0.59, 1.06)	0.69 (0.45, 1.05)

Note. Results in bold indicate significance at a significance level of 0.05.

* ACP: Advance care planning.

## Data Availability

The Health and Retirement Study (HRS) datasets are available in a publicly accessible repository and require registration and/or an agreement with HRS for data access.

## References

[1] Alzheimer’s Association. 2023 Alzheimer’s Disease Facts and Figures. J. Alzheimer’s Assoc 2023, 19, 1598–1695. 10.1002/alz.13016 36918389

[2] Matthews KA; Xu W; Gaglioti AH; Holt JB; Croft JB; Mack D; McGuire LC Racial and ethnic estimates of Alzheimer’s disease and related dementias in the United States (2015–2060) in adults aged 65 years. Alzheimer’s Dement. 2019, 15, 17–24. 30243772 10.1016/j.jalz.2018.06.3063 PMC6333531

[3] Gottesman RF; Albert MS; Alonso A; Coker LH; Coresh J; Davis SM; Deal JA; McKhann GM; Mosley TH; Sharrett AR Associations Between Midlife Vascular Risk Factors and 25-Year Incident Dementia in the Atherosclerosis Risk in Communities (ARIC) Cohort. JAMA Neurol. 2017, 47, 1246–1254. 10.1001/jamaneurol.2017.1658 PMC5710244 28783817

[4] Nelson-Brantley H; Buller C; Befort C; Ellerbeck E; Shifter A; Ellis S Using implementation science to further the adoption and implementation of advance care planning in rural primary care. J. Nurs. Scholarsh 2020, 52, 55–64. 31545557 10.1111/jnu.12513 PMC6953624

[5] Lipnicki DM; Sachdev PS; Crawford J; Reppermund S; Kochan NA; Trollor JN; Draper B; Slavin MJ; Kang K; Lux O Risk Factors for Late-Life Cognitive Decline and Variation with Age and Sex in the Sydney Memory and Ageing Study. PLoS ONE 2013, 8, e65841. 23799051 10.1371/journal.pone.0065841 PMC3683032

[6] Jones K; Birchley G; Huxtable R; Walter T; Dixon J End of Life Care: A Scoping Review of Experiences of Advance Care Planning for People with Dementia. Dementia 2019, 18, 825–845. 27821714 10.1177/1471301216676121

[7] Sudore R; Lum H; You J; Al E Defining advance care planning for adults: A consensus definition from a multidisciplinary Delphi panel. J. Pain Symptom Manag 2017, 53, 821–832.e21. 10.1016/j.jpainsymman.2016.12.331 PMC5728651 28062339

[8] McMahan RD; Tellez I; Sudore RL Deconstructing the complexities of advance care planning outcomes: What do we know and where do we go? A Scoping Review. J. Am. Geriatr. Soc 2021, 69, 234–244. 32894787 10.1111/jgs.16801 PMC7856112

[9] Dalton MA; Langdon TP Estate Planning, 13th ed.; Money Education: St. Rose, LA, USA, 2022.

[10] Mountford W; Dening KH; Green J Advance care planning and decision-making in dementia care: A literature review. Nurs. Older People 2024, 36, 5. 10.7748/nop.2020.e1238 32666719

[11] Ryan T; M-amen K; Mckeown J The advance care planning experiences of people with dementia, family caregivers and professionals: A synthesis of the qualitative literature. Ann. Palliat. Med 2017, 6, 380–389. 28754049 10.21037/apm.2017.06.15

[12] Rahemi Z; Williams CL Does ethnicity matter—Cultural factors underlying older adults’ end-of-life care preferences: A systematic review. Geriatr. Nurs 2020, 41, 89–97. 31320127 10.1016/j.gerinurse.2019.07.001

[13] Ashana DC; D’Arcangelo N; Gazarian PK; Gupta A; Perez S; Reich AJ; Tjia J; Halpern SD; Weissman JS; Ladin K ‘Don’t Talk to Them About Goals of Care’: Understanding Disparities in Advance Care Planning. J. Gerontol. Ser. A Biol. Sci. Med. Sci 2022, 77, 339–346. 33780534 10.1093/gerona/glab091 PMC8824574

[14] Crooks J; Trotter S; Obe RB; Monaghan E; Clarke G How does ethnicity affect presence of advance care planning in care records for individuals with advanced disease? A mixed-methods systematic review. BMC Palliat. Care 2023, 22, 43. 37062841 10.1186/s12904-023-01168-7 PMC10106323

[15] Elk R Partnering with minoritized communities to reduce health disparities: A focus on advance care planning. J. Am. Geriatr. Soc 2023, 71, 2369–2372. 37432098 10.1111/jgs.18491

[16] Rahemi Z; Malatyali A; Wiese LAK; Dye CJ End-of-Life Care Planning in Diverse Individuals Across Age Groups: A Proposed Conceptual Model of Nursing. J. Nurs. Care Qual 2023, 38, 319–326. 36947814 10.1097/NCQ.0000000000000705 PMC10442095

[17] Sonnega A; Faul JD; Ofstedal MB; Langa KM; Phillips JWR; Weir DR Cohort profile: The Health and Retirement Study (HRS). Int. J. Epidemiol 2014, 43, 576–585. 24671021 10.1093/ije/dyu067 PMC3997380

[18] HRS Staff. Sampling Weights: Revised for Tracker 2.0 & Beyond; The Health and Retirement Study: Ann Arbor, MI, USA, 2019.

[19] Winship C; Radbill L Sampling Weights and Regression Analysis. Sociol. Methods Res 1994, 23, 230–257.

[20] Langa KM; Larson EB; Crimmins EM; Faul JD; Levine DA; Kabeto MU; Weir DR A comparison of the prevalence of dementia in the United States in 2000 and 2012. JAMA Intern. Med 2017, 177, 51–58. 27893041 10.1001/jamainternmed.2016.6807 PMC5195883

[21] Clayton JL; Supiano KP; Aruscavage N; Bybee SG; Utz RL; Iacob E; Dassel KB Advance Care Planning in the Context of Dementia: Defining Concordance. Gerontol. Soc. Am 2024, 64, gnae029. 10.1093/geront/gnae029 PMC11106733 38537649

[22] Jacobsen J; Bernacki R; Paladino J Shifting to serious illness communication. J. Am. Med. Assoc 2022, 327, 321–322. 10.1001/jama.2021.23695 34994773

[23] Pettigrew C; Brichko R; Black B; O’Connor MK; Austrom MG; Robinson MT; Lindauer A; Shah RC; Peavy GM; Meyer K Attitudes toward advance care planning among persons with dementia and their caregivers. Int. Psychogeriatr 2020, 32, 585–599. 31309906 10.1017/S1041610219000784 PMC6962575

[24] Gaster B; Larson EB; Curtis JR Advance directives for dementia meeting a unique challenge. J. Am. Med. Assoc 2017, 318, 2175–2176. 10.1001/jama.2017.16473 29114779

[25] Fried TR; Cohen AB; Harris JE; Moreines L Cognitively Impaired Older Persons’ and Caregivers’ Perspectives on Dementia-Specific Advance Care Planning. J. Am. Geriatr. Soc 2021, 69, 932–937. 33216955 10.1111/jgs.16953 PMC8300881

[26] Rahemi Z; Parker V Does culture matter? Young and middle-aged Iranian-American adults’ perspectives regarding end-of-life care planning. Am. J. Hosp. Palliat. Med 2021, 39, 555–561. 10.1177/10499091211036894 34365832

[27] Rahemi Z Planning Ahead for End-of-Life Healthcare among Iranian-American Older Adults: Attitudes and Communication of Healthcare Wishes. J. Cross Cult. Gerontol 2019, 34, 187–199. 31073970 10.1007/s10823-019-09371-x

[28] Koss CS; Baker TA Where There’s a will: The link between estate planning and disparities in advance care planning by white and Black older adults. Res. Aging 2018, 40, 281–302. 29298597 10.1177/0164027517697116

[29] Hong M; Hye E; Kimberly Y; Margaret JJ Facilitators and Barriers for Advance Care Planning Among Ethnic and Racial Minorities in the U.S.: A Systematic Review of the Current Literature. J. Immigr. Minor. Health 2018, 20, 1277–1287. 29124502 10.1007/s10903-017-0670-9

[30] Huang HL; Lu WR; Liu CL; Chang HJ Advance care planning information intervention for persons with mild dementia and their family caregivers: Impact on end-of-life care decision conflicts. PLoS ONE 2020, 15, e0240684. 33052970 10.1371/journal.pone.0240684 PMC7556500

[31] Rahemi Z; Williams CL Development of a dignity-enhancing model of caring for older adults. Int. J. Hum. Caring 2015, 19, 36–42.

[32] Morrison RS; Meier DE; Arnold RM What’s wrong with advance care planning? J. Am. Med. Assoc 2021, 326, 1575–1576. 10.1001/jama.2021.16430 PMC9373875 34623373

